# Open data set of live cyanobacterial cells imaged using an X-ray laser

**DOI:** 10.1038/sdata.2016.58

**Published:** 2016-08-01

**Authors:** Gijs van der Schot, Martin Svenda, Filipe R.N.C. Maia, Max F. Hantke, Daniel P. DePonte, M. Marvin Seibert, Andrew Aquila, Joachim Schulz, Richard A. Kirian, Mengning Liang, Francesco Stellato, Sadia Bari, Bianca Iwan, Jakob Andreasson, Nicusor Timneanu, Johan Bielecki, Daniel Westphal, Francisca Nunes de Almeida, Duško Odić, Dirk Hasse, Gunilla H. Carlsson, Daniel S.D. Larsson, Anton Barty, Andrew V. Martin, Sebastian Schorb, Christoph Bostedt, John D. Bozek, Sebastian Carron, Ken Ferguson, Daniel Rolles, Artem Rudenko, Sascha W. Epp, Lutz Foucar, Benedikt Rudek, Benjamin Erk, Robert Hartmann, Nils Kimmel, Peter Holl, Lars Englert, N. Duane Loh, Henry N. Chapman, Inger Andersson, Janos Hajdu, Tomas Ekeberg

**Affiliations:** 1 Laboratory of Molecular Biophysics, Department of Cell and Molecular Biology, Uppsala University, Husargatan 3 (Box 596), SE-751 24 Uppsala, Sweden; 2 LCLS, SLAC National Accelerator Laboratory, 2575 Sand Hill Road, Menlo Park, California 94025, USA; 3 European XFEL, Albert-Einstein-Ring 19, 22761 Hamburg, Germany; 4 Arizona State University, Physics Department, PO Box 871504, Tempe, Arizona 85287-1504, USA; 5 Center for Free-Electron Laser Science, DESY, Notkestrasse 85, 22607 Hamburg, Germany; 6 I.N.F.N. and Physics Department, University of Rome ‘Tor Vergata’, Via della Ricerca Scientifica 1, 00133 Rome, Italy; 7 Deutsches Elektronen-Synchrotron DESY, Notkestrasse 85, 22607 Hamburg, Germany; 8 ELI beamlines, Institute of Physics, Academy of Sciences of the Czech Republic, Na Slovance 2, 18221 Prague, Czech Republic; 9 Department of Physics and Astronomy, Uppsala University, Lägerhyddsvägen 1, Box 516, SE-751 20 Uppsala, Sweden; 10 MRC Laboratory for Molecular Cell Biology, UCL, Gower St., London WC1E 6BT, UK; 11 Center for Technology Transfer and Innovation, Jozef Stefan Institute, Jamova cesta 39, SI-1000 Ljubljana, Slovenia; 12 ARC Centre of Excellence for Advanced Molecular Imaging, School of Physics, The University of Melbourne, Victoria 3010, Australia; 13 Institut für Optik und Atomare Physik, Technische Universität Berlin, Hardenbergstrasse 36, 10623 Berlin, Germany; 14 Synchrotron SOLEIL, L’orme des Merisiers roundabout of St Aubin, 91190 Saint Aubin, France; 15 Max Planck Advanced Study Group, Center for Free Electron Laser Science, Notkestrasse 85, 22607 Hamburg, Germany; 16 Max-Planck-Institut für medizinische Forschung, Jahnstr. 29, 69120 Heidelberg, Germany; 17 Max-Planck-Institut für Kernphysik, Saupfercheckweg 1, 69117 Heidelberg, Germany; 18 PNSensor GmbH, Otto-Hahn-Ring 6, 81739 Munich, Germany; 19 Max-Planck-Institut Halbleiterlabor, Otto-Hahn-Ring 6, 81739 München, Germany; 20 Max-Planck-Institut für extraterrestrische Physik, Giessenbachstrasse, 85741 Garching, Germany; 21 Ultrafast Coherent Dynamics Group, University Oldenburg, Carl-von-Ossietzky Strasse 9-11, 26129 Oldenburg, Germany; 22 Centre for BioImaging Sciences, National University of Singapore, 14 Science Drive 4 Blk S1 A, Singapore 117546, Singapore; 23 University of Hamburg, Notkestrasse 85, 22607 Hamburg, Germany

**Keywords:** Molecular biophysics, X-rays, Imaging

## Abstract

Structural studies on living cells by conventional methods are limited to low resolution because radiation damage kills cells long before the necessary dose for high resolution can be delivered. X-ray free-electron lasers circumvent this problem by outrunning key damage processes with an ultra-short and extremely bright coherent X-ray pulse. Diffraction-before-destruction experiments provide high-resolution data from cells that are alive when the femtosecond X-ray pulse traverses the sample. This paper presents two data sets from micron-sized cyanobacteria obtained at the Linac Coherent Light Source, containing a total of 199,000 diffraction patterns. Utilizing this type of diffraction data will require the development of new analysis methods and algorithms for studying structure and structural variability in large populations of cells and to create abstract models. Such studies will allow us to understand living cells and populations of cells in new ways. New X-ray lasers, like the European XFEL, will produce billions of pulses per day, and could open new areas in structural sciences.

## Background & Summary

Imaging living cells at resolutions higher than the resolution of optical microscopy is challenging. A dose in excess of hundred million Grays (Gy: Jkg^−1^) is required to reach sub-nanometer resolution on a micron-sized cell, using X-rays or electrons, and no cell can survive this level of irradiation; a dose of only hundred Grays kills most cells^1,2^. What is known about cells today at high resolution comes from dead cells.

Ultra-short and extremely intense coherent X-ray pulses from X-ray lasers offer the possibility to outrun key damage processes^3^ and deliver a molecular-level snapshot of a cell that is alive at the time of image formation^4^ but explodes a few picosecond later^5^. ‘Diffraction-before-destruction’^3,4,6^ has been successfully demonstrated on a wide range of biological samples, including protein nanocrystals^7^, living cells^8^, cell organelles^9^ and virus particles^10^. Recent results also show 3D reconstruction of reproducible virus particles^11^. The data sets described in this paper are from similar ultra-fast imaging experiments.

The ability to measure millions of diffraction patterns in a day at X-ray free-electron lasers (XFELs) offers new avenues for experiments on cells. The femtosecond illumination ‘freezes’ all motion in the sample on the time scale of atomic vibrations. The massive amount of data emerging from XFELs will represent more than just individual projection images of cells. There is a need to develop algorithms to create abstract models of cells from the data, where no individual image gives us complete information but rather nudges the abstraction to describe common features and common internal interactions. Conversely, the data can be used to describe structural variability in populations. With so many images per day, even statistically rare events could be pinpointed and studied. The data sets also offer innovative avenues for data-driven discovery, and helping this effort was one of our motivations in releasing the data sets.

Cyanobacteria were used in this experiment because of their small size and for being remarkably robust. Solitary *C. gracile* cells are between 0.25–0.4 μm in diameter and 0.4–2.4 μm long^12^. The *S. elongatus* cells are similar in diameter but are longer on average by up to a micron. Both species divide symmetrically by binary fission. The two daughter cells separate from each other after reaching the size and shape of the mother cell^13^. We used non-synchronized cell cultures in our studies, undergoing active growth and providing cells in various stages of their cell cycle.

The live cells were delivered into the pulse train of the Linac Coherent Light Source (LCLS) in an aerosol at a reduced pressure using methods developed for studies on giant viruses^10^. This type of sample injection delivers truly isolated samples into the X-ray beam and gives diffraction patterns with practically no background noise. In addition, the contrast between the sample and its surrounding (wet helium gas expanding into a vacuum chamber) is also exceptionally high. Injected cells arrive in random order and are imaged in random orientations. The data sets include images with signal extending beyond 4 nm resolution.

At these wavelengths and the scattering angles of the strongest patterns, a single diffraction pattern contains limited depth information, and this information may be retrieved by a numerical propagation of the complex-valued wave front^4,6,14,15^. There is a need to explore possibilities to extract depth information from the patterns, and a community effort would speed up progress here.

In order to facilitate developments, we present two data records containing a total of 199,000 diffraction patterns from living cells (Data Citation 1), making it the largest freely available X-ray diffraction data set on cells collected at an X-ray FEL. A subset of 11 diffraction patterns from these data sets was used in a recent publication on imaging live cells^8^. We hope the release of these very large data sets will stimulate interest and help software development.

## Methods

### Experimental setup

The experiment was executed using the CFEL-ASG Multi-Purpose (CAMP) instrument^16^, at the AMO end station^17^ of the Linac Coherent Light Source (LCLS)^18^, using an experimental configuration identical to that used in ref. 10. The bandwidth of the LCLS is approximately 0.5%. The length of the electron bunch was ~70 fs (full-duration at half-maximum) and the length of the photon bunch is believed to be shorter. The size of the focal spot was 3 μm×7 μm (full width at half maximum).

The two data records presented in this paper come from two experiments, using different experimental parameters (Table 1). In *Experiment 1* we collected diffraction patterns from *C. gracile* cells. The patterns are presented in data record 1. The photon energy of experiment 1 was 517 eV (2.40 nm wavelength). In *Experiment 2* we collected diffraction patterns from *S. elongatus*, presented in data record 2. The photon energy of experiment 2 was 1,100 eV (1.13 nm).

**Figure 1:**
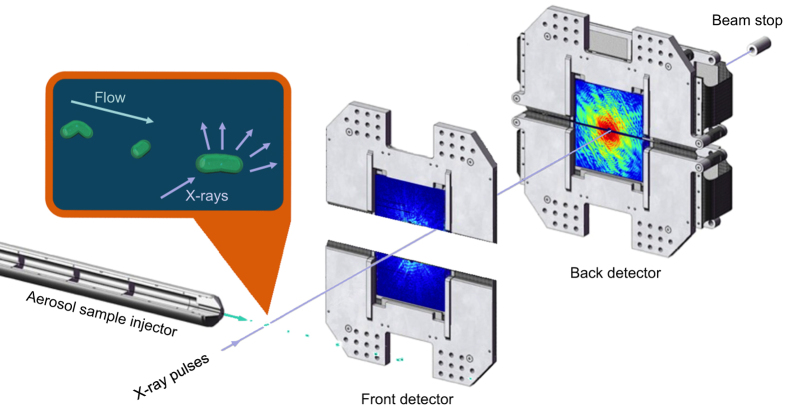
The experimental arrangement. *C. gracile* and *S. elongatus* cells were injected into the pulse train of the LCLS^18^ at 10^−6^ mbar pressure, using an aerosol sample injector built in Uppsala. The cells are in different stages of division, and arrive in random order and are imaged in random orientations. The diffracted signal is recorded on two detector pairs (front detector and back detector). The direct beam passes through an opening between the two detector halves of each detector pair^16^. The opening or gap between the front detector halves is 55.6 mm for experiment 1 (*C. gracile* cells), and 22.8 mm for experiment 2 (*S. elongatus* cells). The gap between the back detector halves is closed for both experiments.

The interaction chamber was equipped with two pairs of pnCCD^16^ X-ray area detectors (front and back detectors), each consisting of two movable detector panels (Fig. 1). The front detector assembly was placed 220 mm from the interaction point, and the back detector assembly at 741 mm in both experiments. The gap between the two front detector panels was 55.6 mm for experiment 1 and 22.8 mm for experiment 2. The gap between the back detector panels was closed in both experiments. The direct beam exited through openings between the two detector halves and was absorbed in a beam dump behind the back detectors. Each detector panel contained 512×1,024 pixels with 75 μm edge lengths and a full-well capacity of 500,000 electrons/pixel, corresponding to 3,500 photons in experiment 1 and 1,600 photons in experiment 2. The read-out rate matched the 120 Hz repetition rate of the LCLS.

### Cells

*Cyanobium gracile* PCC 6307 and *Synechococcus elongatus* PCC 7942 cells were grown in the standard Bg11 medium in batch cultures under constant light. The cell cultures were non-synchronized providing cells in various stages of division. Before the imaging experiments, cells were centrifuged at 6,500 g for 10 min, creating a soft pellet. The pellet was resuspended in 25 mM ammonium acetate, and this buffer exchange was repeated twice to remove salt and contaminants.

### Sample injection

The suspension of live cells was aerosolized with helium in a gas dynamic nebulizer^19^. The aerosols were delivered into the pulse train of the X-ray laser through an aerodynamic lens^20^. This method delivers cells in free flight without substrate or other supporting medium, thereby minimizing background scattering, and can produce millions of exposures per day. Most of the nebulizing gas, and vapours of the volatile buffer were pumped away through a differential pumping stage.

### Data recording

**Figure 2:**
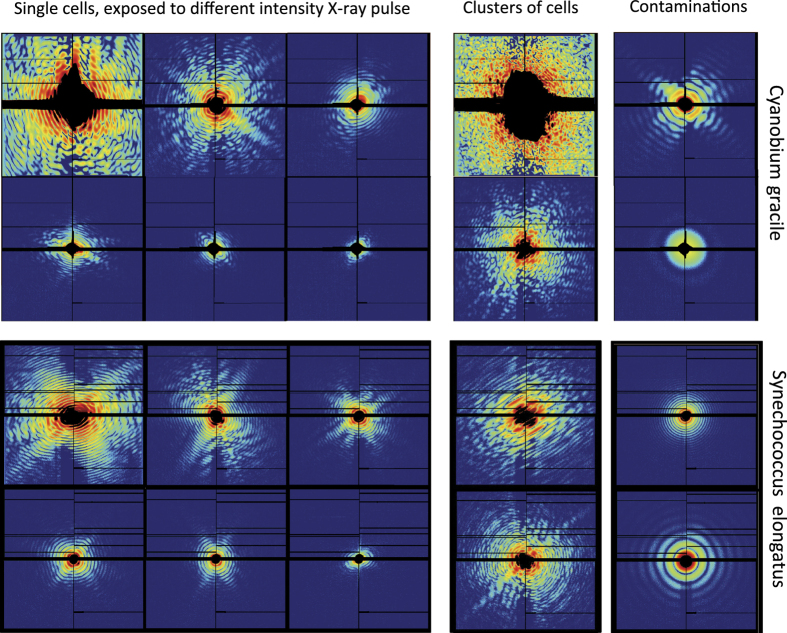
Compilation of representative sampling of diffraction patterns from both experiments. Ten representative diffraction patterns from each data record were selected. Both data sets contain diffraction patterns from single living cells, multiple cells, large clusters of cells, as well as from contaminants such as spherical droplets, or virus-like particles (possibly being an earlier injected sample). All patterns are normalized individually; dark blue is no scattered signal, dark red is most intense signal in the pattern.

We recorded a variety of diffraction patterns, originating from single cells, clusters of cells, droplets of buffer or contaminants. The patterns have a large variation in recorded intensity, depending on X-ray pulse-intensity, where in the pulse the particle was hit, and the size of the particle. Figure 2 shows a representative set of diffraction patterns from both experiments.

### Data preprocessing

We also supply a minimally preprocessed dataset, which includes only diffraction patterns with significant scattered signal (199,000 out of 540,000 patterns). The preprocessing included generation and subtraction of calibration levels, the masking of faulty pixels, the application of the experimental geometry, and a background subtraction. Frames were considered hits if more than 300 pixels record a value above 45 arbitrary detector units (ADU) for experiment 1, and 4,000 pixels recording a value above 45 ADU for experiment 2. The increased threshold in experiment 2 compensates for a stronger background scattering present in experiment 2. All preprocessing steps were done automatically using the *Cheetah* software package^21^. The Cheetah configuration files, calibration data, the bad pixel masks, and the respective geometry files are also included into the Data records (Tables 2 and 3).

## Data Records

### Data record 1

Data record 1 contains the raw and preprocessed data of 473,447 snapshots from *C. gracile* measured during 77 min of beam-time. The X-ray photon energy was 512 eV and both front and back detector panels were included. The snapshots include blank shots, hits of contaminants, and hits of single and multiple *C. gracile* cells in random orientation and in random stages of the cell cycle, exposed to different pulse intensities. We estimate the hit ratio of *C. gracile* cells to be 41% (192,370 diffraction patterns).

The raw data is provided in extended tagged container (XTC) format, and the preprocessed data is provided in CXI format. Both are available for download from the Coherent X-ray Imaging Data Bank (CXIDB)^22^.

### Data record 2

Data record 2 contains the raw and preprocessed data of 66,442 snapshots from *S. elongatus* measured during 9 min of beam-time. The X-ray wavelength was 1,100 eV and both front and back detector panels were included. The snapshots include blank shots, hits of contaminants, and hits of single and multiple *S. elongatus* cells in random orientation and in random stages of the cell cycle, exposed to different pulse intensities. We estimate the hit ratio of *S. elongatus* cells to be 10% (6394 diffraction patterns).

The raw data is provided in XTC format, and the preprocessed data is provided in CXI format, both available at the CXIDB (Data Citation 1).

## Technical Validation

### Viability of cells

In ref. 8 we show that the injection method was not disruptive to the cells and that the cells were alive at the moment of exposure to the X-ray pulse.

### Contamination

We have observed contaminants to be present in each data set, i.e., spherical droplets, and virus-like particles. The former is a common artifact from the injection method, and the latter is most likely samples injected earlier that remained in the pipeline. Both contaminants are easily distinguished by their diffraction patterns (see Fig. 2).

### Reconstruction validation

It is shown in ref. 8 that diffraction patterns from this data set can be phased and that the resulting electron densities are matching expectations.

## Usage Notes

The data is available in CXI format^22^ (Table 4). CXI uses the HDF5 format^23^ which is readable in many computational environments such as matlab, python and C.

## Additional Information

**How to cite this article:** van der Schot, G. *et al.* Open data set of live cyanobacterial cells imaged using an X-ray laser. *Sci. Data* 3:160058 doi: 10.1038/sdata.2016.58 (2016).

## Supplementary Material



## Figures and Tables

**Table 1:** Experimental setup.

**Sample**	**Experiment 1**	**Experiment 2**
Cell type	*Cyanobium gracile*	*Synechococcus elongatus*
Source Parameters		
End station	AMO	AMO
Repetition rate	120 Hz	120 Hz
Pulse duration	~70 fs	~70 fs
Photons per pulse	~1.5×10^13^ (1.26 mJ)	1.2×10^13^ (2.18 mJ)
Optical efficiency	15%	15%
Bandwidth	0.5%	0.5%
Photon energy	517 eV (2.4 nm)	1,100 eV (1.13 nm)
Focal size	3 μm×7 μm	3 μm×7 μm
Flux in the focus	1.1×10^11^ photons/μm^2^	8.6×10^10^ photons/μm^2^
Detector Properties		
Distance from interaction point (front detector)	220 mm	220 mm
Gap size (front detector)	55.6 mm	22.8 mm
Full-well capacity (front detector)	3,500 photons	1,600 photons
Distance from interaction point (back detector)	741 mm	741 mm
Gap size (back detector)	0 mm	0 mm
Full-well capacity (back detector)	3,500 photons	1,600 photons
This table describes the experimental parameters used in experiment 1 and experiment 2. The sample, the source parameters, and the detector properties.		

**Table 2:** Deposited data and configuration files.

**Data type**	**Example filename**	**File format**
Experimental Data		
Diffraction data (all exposures)	e54-r0207-s00-c00.xtc	XTC
Preprocessed data (only hits)	preprocessed_hits_exp1-r0207.cxi	CXI
Calibration data (dark run)	calibration_data_back_detector_exp1.h5	Hdf5
Preprocessing		
Cheetah initialization file (generate calibration data)	cheetah_calibration_exp1.ini	Text
Cheetah configuration file (generate calibration data)	psana_calibration_exp1.cfg	Text
Calibration data (*back* detector)	calibration_data_back_detector_exp1.h5	Hdf5
Calibration data (*front* detector)	calibration_data_front_detector_exp1.h5	Hdf5
Cheetah initialization file (preprocessing)	cheetah.ini	Text
Cheetah configuration file (preprocessing)	psana.cfg	Text
Bad pixel map (*back* detector)	badpixelmap_back_detector_exp1.h5	Hdf5
Bad pixel map (*front* detector)	badpixelmap_front_detector_exp1.h5	Hdf5
Experimental geometry (*back* detector)	geometry_back_detector_exp1.h5	Hdf5
Experimental geometry (*front* detector)	geometry_front_detector_exp1.h5	Hdf5
This table describes the files deposited on the CXIDB under accession number ID-37. ID-37 consists of two data records, from two experiments. Both records contain all these files.		

**Table 3:** Description of the experimental data sets.

**Records**	**Cell species**	**Data Type**	**Run number**	**Size (# exposures)**	**Hit ratio**	**Duration run**
Data Record 1	No sample	Calibration data	r0206	885	N.A.	1 min
	*C. gracile*	Diffraction data	r0207	195,491	42%	27 min
	*C. gracile*	Diffraction data	r0210	67,685	39%	9 min
	*C. gracile*	Diffraction data	r0212	4,222	46%	1 min
	*C. gracile*	Diffraction data	r0214	206,049	40%	29 min
Data Record 2	No Sample	Calibration data	r0140	2,597	N.A.	2 min
	*S. elongatus*	Diffraction data	r0142	66,442	10%	9 min
This table describes the experimental data of the two data records; the species of cyanobacteria imaged, the data type, the run number of the data sets, the size of the data set, the hit ratio, and the duration of the run.						

**Table 4:** Examples of data stored in the cxi format.

**Description**	**Location descriptor**
Preprocessed back detector data of hits	/entry_1/image_1/data
Preprocessed front detector data of hits	/entry_1/image_2/data
Photon energy of hits	/LCLS/photon_energy_eV
Number of lit pixels in hits	/cheetah/event_data/peakNpix
The examples in this table summarize the structure of the cxi files deposited under ID-37. There are three main groups in the cxi format: *LCLS*, *Cheetah*, and *entry_1*. *LCLS* contains various experimental parameters, such as the photon energy for each exposure (photon_energy_eV). *Cheetah* contains cheetah-calculated variables, for example the number of lit pixels (peakNpix). *Entry_1* contains preprocessed front and back detector readouts. It might be useful to use the number of lit pixels to sort your data for instance, or perhaps some of the experimental parameters could be useful for as sort as well. For a detailed description of the data format see ref.22.	
